# Expanding the Genetic and Phenotypic Spectrum of Kearns-Sayre Syndrome: A Case Report

**DOI:** 10.7759/cureus.84293

**Published:** 2025-05-17

**Authors:** Christian Messina

**Affiliations:** 1 Neurology, Azienda Sanitaria Provinciale Catania, Catania, ITA

**Keywords:** chronic progressive external ophthalmoplegia, cpeo, kearns-sayre syndrome, mitochondrial disorders, neuromuscular diseases, oligoclonal band (ocb)

## Abstract

Chronic progressive external ophthalmoplegia (CPEO) is a mitochondrial disorder characterized by progressive bilateral ptosis and symmetric ophthalmoparesis. When CPEO is associated with pigmentary retinopathy, cardiac conduction defects, endocrine abnormalities, muscle weakness, and other neurological impairments, it defines Kearns-Sayre syndrome (KSS), most commonly caused by a single large-scale mitochondrial DNA (mtDNA) deletion. We report a case of a 34-year-old man with a three-year history of progressive bilateral ptosis. A muscle biopsy from the left vastus lateralis revealed cytochrome c oxidase-deficient fibers (COX-negative). mtDNA analysis revealed a novel single large-scale deletion detected in muscle tissue. This deletion has not been previously reported in the scientific literature and led to a diagnosis of KSS. Additionally, cerebrospinal fluid analysis revealed the presence of oligoclonal bands, a finding not previously described in KSS. The deleted mtDNA region includes *ND4*, *ND5*, and *ND6* genes, which encode subunits of NADH dehydrogenase. These genes are implicated in various biological functions, including mitochondrial energy production, seizure susceptibility, and inflammatory processes.

## Introduction

Chronic progressive external ophthalmoplegia (PEO, or CPEO) is a mitochondrial pathological condition characterized by progressive bilateral ptosis and symmetric reduction in ocular motility [1]. It can occur as an isolated oculomotor dysfunction or in association with other systemic symptoms, proximal muscle weakness, and other biochemical features, such as elevation in serum creatine kinase (CK), lactate dehydrogenase (LDH), lactate, and alanine, leading to the definition of “CPEO plus syndrome” [2]. Such a condition can be identified in Kearns-Sayre syndrome (KSS), which is characterized by the occurrence of CPEO, pigmentary retinopathy, cardiac conduction disturbances, endocrine alterations, muscle weakness, and other neurologic impairment. The most frequent causal molecular defects include sporadic single large-scale mitochondrial DNA (mtDNA) deletions, even if maternally inherited point mtDNA mutations and autosomal inheritance cases have been described [3,4]. To the best of our knowledge, we describe the first known case of CPEO associated with both a previously unreported 3844-bp mtDNA deletion (m.10759_14602del) and the unexpected presence of oligoclonal bands in the cerebrospinal fluid.

## Case presentation

In February 2022, a 34-year-old man reported a three-year history of progressive bilateral drooping of the upper eyelid associated with visual impairment at night and in a poorly illuminated environment. His family history was characterized by consanguinity between his paternal grandparents (first cousins). He experienced sleep seizures between 12 and 18 years of age, treated with valproic acid, and was diagnosed with keratoconus at the age of 20 years, treated with corneal cross-linking and then with corneal transplant. At the neurological examination, he presented with severe (>4 mm) bilateral upper eyelid ptosis, adduction deficit of the right eye and impaired abduction of both eyes, a chin-up compensatory position, and frontal muscle weakness. Repetitive nerve stimulation (RNS) was unremarkable, whereas single-fiber electromyography (SFEMG) revealed the presence of abnormal jitter in the right eye, as per the protocol described by Sciacca et al. [5]. Blood tests only revealed a mild elevation in gamma-glutamyl transferase (GGT), with normal CK and LDH. Blood tests are reported in Table 1. CSF analysis was characterized by increased total proteins and albumin and identification of an identical number of oligoclonal bands in both CSF and serum (pattern 4) [6]. Table 2 presents the CSF results. Electroretinogram (ERG) resulted in bilateral retinopathy with impaired P50 latencies. Ophthalmologic evaluation showed the presence of bilateral retinal dystrophy and alteration in retinal pigment epithelium above the foveal region, confirming the suspicion of retinopathy. Cardiac evaluation highlighted mild left ventricular hypertrophy, non-significant anterior pericardial detachment, and mild tricuspid regurgitation. A left vastus lateralis muscular biopsy was performed, which revealed deficient staining for cytochrome c oxidase (COX-negative fibers). Hematoxylin-eosin staining did not reveal fiber size variability, but isolated hypotrophic fibers were observed. A single fiber displayed subsarcolemmal accumulation of purple material. No central nuclei were detected. Gomori trichrome staining did not show increased endomysial or perimysial connective tissue. One ragged red fiber was observed. Oxidative enzyme reactions (nicotinamide adenine dinucleotide tetrazolium reductase (NADH-TR), COX, succinate dehydrogenase (SDH), COX-SDH) revealed subsarcolemmal mitochondrial rims, more prominent with NADH. Several COX-negative fibers were seen, which appeared blue on COX-SDH staining. Acid and alkaline ATPase staining showed a marked predominance of type 2 fibers forming large homotypic clusters. Periodic acid-Schiff (PAS) staining for glycogen was within normal limits. Oil Red O staining for lipids was within normal limits. Lysosomal acid phosphatase staining was within normal limits. Mitochondrial genome sequencing performed on DNA extracted from a muscle biopsy of the vastus lateralis revealed a single 3844-base pair deletion in the mtDNA (m.10759_14602del), affecting approximately 47% of the mtDNA molecules in the analyzed muscle tissue sample, still not been described in scientific literature. Amplification was performed by long-range polymerase chain reaction (LR-PCR), while PCR-free sequencing was performed using Oxford Nanopore technology (Oxford Nanopore Technologies Ltd., Oxford, United Kingdom) (for breakpoint definition and heteroplasmy quantification), with reference sequence NC_012920. Based on results from the diagnostic workup, including clinical and laboratory features (PEO, retinopathy, structural cardiac alteration without conduction defects, abnormal jitter in SFEMG, myopathy, presence of RFF COX negative), a diagnosis of KSS was confirmed.

**Table 1:** Blood tests with their results during hospitalization Hb: hemoglobin; MCV: mean corpuscular volume; WBC: white blood count; CRP: C-reactive protein; LDH: lactate dehydrogenase; AST: aspartate aminotransferase; ALT: alanine aminotransferase; GGT: gamma-glutamyl transferase; CPK: creatine phosphokinase; TSH: thyroid-stimulating hormone; ANA: antinuclear antibodies; ENA: extractable nuclear antigens; IgM: immunoglobulin M; IgG: immunoglobulin G; HSV1: herpes simplex virus 1; HSV2: herpes simplex virus 2; VZV: varicella-zoster virus; CMV: cytomegalovirus; EBV: Epstein-Barr virus; VCA: antiviral capsid antigen; VDRL: Venereal Disease Research Laboratory; FTA-ABS: fluorescent treponemal antibody absorption; IGRA: interferon-gamma release assay

Lab tests	Results	Normal values
Hb	11.5 g/dL	10.5-13.5 g/dL
MCV	88 fL	80-100 fL
WBC	5.8 x 10^9^/L	4.0-11.0 x 10^9^/L
Neutrophils	3.7 x 10^9^/L	1.8-7.5 x 10^9^/L
Lymphocytes	2.1 x 10^9^/L	1.0-4.0 x 10^9^/L
CRP	0.5 mg/L	<5 mg/L
LDH	197 U/L	135-225 U/L
Sodium	137 mmol/L	135-145 mmol/L
Potassium	3.7 mmol/L	3.5-5.3 mmol/L
Glucose	95 mg/dL	69-105 mg/dL
Urea	5.2 mmol/L	2.5-7.8 mmol/L
Creatinine	0.82 mg/dL	<1.10 mg/dL
AST	13 U/L	8-33 U/L
AST	37 U/L	15-40 U/L
GGT	123 U/L	8-61 U/L
Lipase	65 U/L	14-72 U/L
Total amylase	98 U/L	30-118 U/L
CPK	156 U/L	29-168 U/L
TSH	3.7 μU/mL	2-10 μU/mL
ANA	1:40	<1:80
ENA screening	Negative	Negative
Anti-gangliosides IgM antibodies	Negative	Negative
Anti-gangliosides IgG antibodies	Negative	Negative
HSV1 IgM	8.9 U/mL	<20 U/mL
HSV1 IgG	23 U/mL	<20 U/mL
HSV2 IgM	6.5 U/mL	<20 U/mL
HSV2 IgG	12.5 U/mL	<20 U/mL
VZV IgM	9.2 U/mL	<25.5 U/mL
VZV IgG	26.2 U/mL	<25.5 U/mL
CMV IgM	0.5 U/mL	<18 U/mL
CMV IgG	2.3 U/mL	<18 U/mL
EBV VCA IgM	22 U/mL	<20 U/mL
EBV VCA IgG	12 U/mL	<35.9 U/mL
Toxoplasma IgM	1.2 IU/mL	<9 IU/mL
Toxoplasma IgG	7.3 IU/mL	<9 IU/mL
VDRL	Negative	<1:16
FTA-ABS	Negative	Negative
IGRA	Negative	Negative

**Table 2:** CSF analysis CSF: cerebrospinal fluid; IgG: immunoglobulin G; OCBs: oligoclonal bands

CSF analysis	Results	Normal values
Proteins	323 mg/L	135-500 mg/L
Glucose	69 mg/dL	<75 mg/dL
Cells	1/µL	<5/µL
IgG	63 mg/dL	-
IgG index	0.52	<0.7
OCBs	Four OCBs in both CSF and serum	No OCBs
Anti-gangliosides IgM antibodies	Negative	Negative
Anti-gangliosides IgG antibodies	Negative	Negative

## Discussion

CPEO is a mitochondrial disease characterized by chronic progressive worsening in ocular motility impairment and bilateral ptosis. It is the most common manifestation in mitochondrial myopathies, occurring in two-thirds of patients, mostly affecting young adults between 18 and 40 years of age [7]. Due to its clinical presentation, an accurate differential diagnosis is needed in order to exclude thyroid ophthalmopathy, dystrophies, myasthenia gravis, Lambert-Eaton syndrome, and paraneoplastic brainstem encephalitis [7]. Ptosis is defined by an abnormally low-positioned upper eyelid, whose severity ranges from mild (if the cornea is covered up to 2 mm) to moderate (if the cornea is covered 3 to 4 mm) or severe (if the cornea is covered over 4 mm) [8]. Bilateral ptosis, together with weakness of the frontalis muscle and compensatory chin elevation, defines the characteristic Hutchinson's triad, observed in our patient [7]. Except for rare cases of unilateral ptosis, PEO rarely causes diplopia due to its symmetry and progressive course, which allows the instauration of central compensatory strategies [1,4]. The association with other mitochondrial symptoms, unless satisfying diagnostic criteria for defined mitochondrial diseases, leads to the denomination of CPEO plus [9]. CPEO plus may be characterized by ataxia, migraine, epilepsy, optic atrophy, cataract, retinal degeneration, paraparesis, hypothyroidism, hyperlipidemia, cardiomyopathy, and neuropathy, as well as by hypersomnia, facial tics, glaucoma, basal ganglia calcification, hepatopathy, diverticulosis, chronic gastritis, hyperhidrosis, scalp atheromasias, and psoriasis [7]. In this view, CPEO, CPEO plus, and KSS could be seen as a spectrum disorder. The patient described in the case presented CPEO, cardiomyopathy, epilepsy, retinal dystrophy, and keratoconus, satisfying the diagnostic criteria for KSS, but without satisfying the criteria for mitochondrial encephalomyopathy, lactic acidosis, and stroke-like episodes (MELAS) syndrome, since the elevation in serum lactate and focal neurological episodes were not observed [1,10]. Further, serum CK analyses, blood glucose, and serum creatinine in blood tests can be normal or mildly impaired in CPEO and KSS [1-3]. An increase in CSF proteins was described in KSS, but not in CPEO, and the presence of oligoclonal bands was never described in both conditions, differently from our patient [10]. Mitochondrial abnormalities in CPEO and KSS are found in 95% of muscle biopsies, such as subsarcolemmal accumulation of red-staining mitochondria on Gomori trichrome stain (ragged-red fibers), increased expression of SDH, an enzyme that is encoded entirely by nuclear DNA (ragged-blue fibers), or deficient staining for cytochrome c oxidase (COX-negative fibers), as in our patient [1-4]. CPEO is characterized by genetic heterogeneity. It is sporadic in 50% of cases, due to a single large de novo mtDNA deletion detected by Southern blot or PCR techniques and typically not transmitted to offspring [1,4,11]. Such an mtDNA deletion can present with wide phenotypic expression ranging from CPEO to KSS to Pearson syndrome [1]. Recent studies have shown that the size and location of the single deletion may predict the clinical phenotype, with larger deletions associated with more severe disease [1,11]. The other half of CPEO cases is inherited through autosomal dominant (AD), autosomal recessive (AR), or maternal transmission [4]. AD PEO is mostly caused by mutation of *POLG1*, *POLG2*, *ANT1*, *TWINKLE*, *RRM2B*, *DNA2* and *OPA1*, with *POLG1* as the most common [1]. AR PEO is caused by mutation of the *TYMP*, *POLG1*, *DGUOK*, *TK2*, *MGM1*, and *RNASEH1* genes [1]. Maternal transmission is due to point mutations of mtRNALeu, mtRNAGln, mtRNAAla, mtRNATyr, mtRNALys, mtRNAAsn, mtRNAIle, and mtRNAPro [1]. The mutation found in our patient is a single deletion of 47% of the molecules of the mtDNA of 3844 base pairs (m.10759_14602del). Such a mutation has never been described in scientific literature or in MITOMAP, the mutation mtDNA database, but can be assumed to be pathogenic. This deletion involves *ND4*, *ND5,* and *ND6* mitochondrial genes, which encode, respectively, for NADH dehydrogenase types 4, 5, and 6. These enzymes constitute the NADH dehydrogenase complex or Complex I (Ubiquinone), whose deletion is described in Leber’s hereditary optic neuropathy (LHON) and in KSS [12], thus explaining the clinical phenotype. However, mutation in *ND4* is also described in mesial temporal lobe epilepsy (MTLE) [13], and mutation in *ND5* and *ND6* in Leigh’s syndrome [14], which could explain the occurrence of seizures at a juvenile age in our patient. Finally, some papers highlighted that genetic alterations in Complex I can be associated with the development of keratoconus [15,16], diagnosed in our patient. A peculiar finding in the diagnostic workup of our patient was the identification of identical oligoclonal bands in CSF and serum, which has not been previously described in CPEO and KSS. It has been shown that NAD+/NADH is a critical chemical intermediate acting as an enzyme cofactor in redox reactions and regulating a large spectrum of cellular functions, such as energy metabolism, DNA repair, regulation of the epigenetic landscape, and inflammation [17]. Intriguingly, four novel mutations in the mitochondrial *ND4* gene have been described in patients with multiple sclerosis, allowing us to hypothesize a possible inflammatory theory in mitochondrial diseases such as KSS [18].

## Conclusions

This case report highlights the marked heterogeneity of disorders within the CPEO spectrum, including KSS, which can manifest with a broad range of clinical, biochemical, radiological, and molecular features. The phenotypic variability observed in these mitochondrial disorders continues to pose diagnostic challenges, particularly in the early stages of disease or in atypical presentations. Of particular interest is the detection of oligoclonal bands in the CSF, a finding not previously reported in KSS to our knowledge. While oligoclonal bands are typically associated with inflammatory or autoimmune central nervous system conditions, their presence in this case raises intriguing questions regarding a potential, yet unexplored, immunological component in the pathogenesis of mitochondrial diseases. The immune system’s role in secondary processes, whether as a consequence of mitochondrial dysfunction or as a contributor to neuroinflammation, deserves further attention, warranting further investigation into their pathogenic and diagnostic significance.
